# Evaluation of the completeness of a medical record dataset linked to administrative claims through tokenization in patients with Hemophilia B

**DOI:** 10.1371/journal.pone.0355051

**Published:** 2026-09-08

**Authors:** Anna Stachel Kane, Diana Madalina Stan, Darren Jeng, Yong Chen

**Affiliations:** 1 Pfizer Inc, United States of America; 2 Pfizer Inc, Bucharest, Romania; Cleveland Clinic Lerner Research Institute, UNITED STATES OF AMERICA

## Abstract

Real-world evidence (RWE) is increasingly used to inform healthcare delivery and expedite access to new therapies, but its value depends on the quality and completeness of information in real-world data (RWD) sources. Tokenization technology enables the integration of patient data from multiple sources, facilitating the development of a comprehensive overview of the patient journey. This study evaluated the accuracy of linked electronic health record (EHR) and insurance claims data for 93 patients with hemophilia B (HB). Tokenization enables secure linkage of patient data from multiple sources by replacing personal identifiers with encrypted tokens, allowing integration while preserving privacy. We assessed the resulting linkage accuracy by comparing hemophilia-related documentation (proportion of patients with matches for hemophilia medication and laboratory results) and patient location (ZIP code). We also evaluated the completeness of these two linked datasets of additional select variables. Tokenization achieved up to 98% linkage accuracy. Completeness varied by data source: EHRs captured 89–100% of key clinical variables, which include disease severity and laboratory values, while claims data ranged from 44–100%, which include healthcare utilization and cost. Full matches were observed in 12% of medication records and 2% of factor laboratory results, highlighting availability in cross-source completeness. Tokenization proved feasible and reliable in a rare disease setting. The small cohort size and rarity of HB enabled manual validation and reduced the risk of erroneous linkages. Findings support the use of tokenization to create composite datasets that integrate clinical and utilization information to provide richer data, thus enhancing the quality of RWE studies in rare diseases. The study’s findings are also limited by the small sample size, which may affect generalizability. However, this study provides a foundational description and provides valuable methodological insights into tokenization accuracy and data completeness across EHR and claims datasets for RWE generation.

## Background

### Real-World Data/Real-World Evidence (RWD/RWE)

RWE studies and applications such as tokenization are increasingly being utilized to improve healthcare delivery and increase the speed of patient access to new drugs [1,2]. Studies utilizing RWD must include thorough investigation of the transparency of RWD collection, the representativeness of the data, and potential biases before drawing any conclusions about the research questions [3].

The United States (US) Food and Drug Administration (FDA) defines RWD in the medical and healthcare field as “data relating to patient health status and/or the delivery of health care routinely collected from a variety of sources” [2]. Some examples of RWD include EHRs, medical and pharmacy insurance claims data, and product and disease registries [2]. EHR systems are used and maintained by healthcare systems to collect and store medical information, comprising of interactions between patients and healthcare providers [4]. EHRs are used to capture a variety of medical information from patients to manage their care and document clinical workflow across various clinical care sites (i.e., hospital, doctor’s office, urgent care, physical therapy) [4,5]. EHRs may contain different types of patient-level variables, such as demographics, diagnoses, problem lists, medications, procedures, vital signs, and laboratory results [5]. Claims data, often referred to as administrative data, represent a large-scale form of electronic records. These databases mainly compile billing and insurance details, but also include information from millions of healthcare interactions, including doctor appointments, prescriptions and other communications between patients and providers [6,7].

In healthcare research, tokenization is a novel way to link RWD sources while preserving patient privacy [8,9]. Tokenization replaces personally identifiable or protected health information (PII/PHI) with unique encrypted tokens, enabling secure linkage of patient data across multiple datasets without exposing individual identities [8,10]. Examples of commonly used PII to create tokens include first name, last name, ZIP code, DOB, and Social Security Number (SSN) [9,11,12]. The researcher can only determine whether two tokens correspond to the same individual based on the matching algorithm applied across datasets, which typically relies on probabilistic rather than deterministic linkage to account for data variation across sources [9,13]. Patient tokens then enable connections between fragmented health data across multiple data platforms to be connected to form a longitudinal view of the patient’s journey [9,13]. Tokenization holds great promise, however, the accuracy of linkages requires further study, with researchers carefully considering the completeness and stability of selected PII elements [9,11]. A recent systematic literature review identified five articles that assessed the effectiveness of privacy-preserving record linkage (PPRL) in accurately matching and deduplicating healthcare records in the US using encrypted PII (first and last names, DOBs and SSNs) [11]. Combinations involving identifiers like names and DOBs were precise, but missed potential links due to data inconsistencies such as misspelled names or name changes [11]. Similarly, combinations of SSNs, although precise, missed even more potential links as SSNs are not currently well captured in the healthcare data [11]. The review showed that using multiple combinations of encrypted PII to find links or deduplicate records was the most precise and accurate method with the highest match rate, while keeping patient data private [11]. A recent study showed that population linking stabilized when at least seven unique encrypted tokens, each derived from combinations of demographic identifiers such as name, DOB, sex, and address, were used, improving linkage precision while minimizing false positives [14].

To our knowledge, this is the first study to evaluate both the accuracy of tokenization technology and the completeness of linked EHR and claims data in a HB population. Because HB is rare, the likelihood of coincidental patient overlap is low, making manual record review more feasible. This contrasts with common prevalent conditions like COVID-19, where large datasets and shared identifiers increase the risk of tokenization errors and make manual validation impractical.

### Tokenization in Healthcare

Tokenization has gained prominence in healthcare as a secure method for protecting sensitive patient data while enabling its use in research, particularly in RWE studies [12]. This approach supports longitudinal studies, enhances interoperability, and complies with privacy regulations such as The Health Insurance Portability and Accountability Act (HIPAA) and General Data Protection Regulation (GDPR) [12,15].

Originally developed for financial data protection, tokenization now plays a critical role in healthcare, enabling secure data sharing for population health management, clinical research, and Health Economics and Outcomes Research (HEOR) [16,17]. Companies like Datavant have advanced this technology by addressing data quality issues with standardization methods, ensuring accurate linking and improving the reliability and security of linked datasets [12,18].

Tokenization benefits stakeholders across the healthcare ecosystem by enabling providers to track clinical progress, payors to analyze patient behavior, and regulators to ensure transparency in data usage [12]. Despite its promise, tokenization’s application in healthcare remains under-researched, with limited studies exploring its accuracy and effectiveness [9,18]. Further validation is necessary to address gaps, such as data completeness and standardization, and to optimize its use in rare disease populations, where each link is valuable [9,12,18,19].

### Hemophilia B

RWE research in HB presents unique challenges due to limitations in claims data and the rarity of the condition. Critical variables such as annualized bleed rates and prescription fill patterns are often missing or incomplete in claims datasets [20,21]. Additionally, claims data typically do not distinguish between prophylactic and on-demand treatment regimens or disease severity, which are essential for understanding disease management in HB.

The rarity of HB further complicates research efforts, as it is difficult to identify a sufficiently large and representative population that includes both structured and unstructured data. However, linking EHR with claims data offers a promising solution. This approach enables the construction of a more comprehensive dataset that integrates clinical details with healthcare utilization metrics, thereby enhancing the ability to assess treatment patterns, disease burden, and unmet needs.

Before implementing studies using linked datasets, it is essential to validate the tokenization process for accuracy and completeness. This study demonstrated that tokenization can be reliably applied in a rare disease context, supporting data integrity and the broader use of linked RWD in rare disease research.

HB is an ideal use for evaluating various RWD sources, being a rare condition that is less susceptible to erroneous linkages via tokenization [22,23]. Its low prevalence allows for manual review and validation of all patient records, providing a unique opportunity to directly assess linkage accuracy, something less feasible in larger, more heterogeneous disease populations. While previous tokenization studies, such as Tyagi et al. 2025, have primarily focused on large, heterogeneous disease populations (e.g., oncology or diabetes), our study addressed a critical gap by demonstrating the feasibility and accuracy of tokenization in a small, rare disease setting [11,24–26]. By confirming that reliable linkages can be achieved even within small cohorts, our findings contribute to improving tokenization standards applicable to both rare and more prevalent conditions.

Hemophilia is a rare inherited bleeding disorder characterized by a deficiency or malfunction of clotting factor. HB is the second most common type of hemophilia, accounting for about 15–20% of hemophilia cases [27]. In the US, there are between 30,000–33,000 patients with hemophilia [28]. Laboratory tests such as activated partial thromboplastin time (APPT) and prothrombin time (PT) are routinely used to diagnose, monitor and manage hemophilia by assessing the efficiency of the blood clotting process [29]. For individuals with HB and a severe bleeding phenotype, the standard of care is prophylactic treatment (regularly scheduled) with infusions of recombinant Factor IX therapy [30,31]. Recent advancements in treatment include extended half-life Factor IX products, anti-tissue factor pathway inhibitor (anti-TFPI), which reduce the frequency of infusions, and gene therapy, which aims to provide a long-term or potentially curative solution by introducing a functional copy of the Factor IX gene into the patient’s cells [32,33].

Research on HB faces significant challenges due to the limited sample sizes and fragmented data across sources [34,35]. This study provides direct value to the HB population by demonstrating the feasibility and utility of token-based linkage across EHR and claims datasets. Claims data often lack critical details, such as disease severity, minor bleeds, and laboratory results, while EHRs often omit comprehensive information on healthcare utilization, including hospitalizations and costs [36,37]. These gaps hinder a complete understanding of the patient journey and unmet needs. By linking claims and EHR data, our approach enables a more complete and longitudinal view of patient care, offering deeper insights into treatment patterns, healthcare utilization, and disease burden [15,38,39]. Given the rarity of HB, the resulting combined dataset was small enough to allow for manual validation, ensuring high data fidelity. Moreover, the low probability of duplicate identifiers in this population, due to its demographic characteristics, enhanced the accuracy of token matches. These factors contribute to a high-quality linked dataset that can support more precise RWE generation, inform clinical decision-making, and guide policy development for rare disease management.

### Electronic health records and insurance claim databases

In healthcare research, RWD is essential for understanding disease patterns, evaluating treatment effectiveness, and assessing outcomes [1,40]. The two primary sources of RWD are EHRs and claims data [41]. Each type of data offers unique benefits and challenges, influencing how useful they are for various research purposes [2,42].

EHRs are comprehensive digital records that contain a variety of patient health data. These records typically include PII like the patient’s name, age and gender, as well as detailed clinical information [12]. The clinical data includes medical history, immunizations, laboratory test results, radiology images, medications, treatment plans, and physician’s notes [43,44]. EHRs also track diagnoses, allergies, and any surgical procedures the patient has undergone [43]. Essentially, EHRs serve as an ongoing record of a patient’s healthcare experience, facilitating coordination among different healthcare providers and ensuring that the patient’s health information is centralized and accessible [44]. Over the past decade, the adoption of EHRs has grown substantially following initiatives such as the US ‘Meaningful Use Program’ introduced in 2011 [45]. This program incentivized healthcare providers to adopt and integrate EHRs to enhance patient care [46]. As a result, EHRs are now widely incorporated into clinical workflows, supporting the use of RWD in research [46]. EHRs provide structured and easily accessible data that help researchers analyze patient outcomes and treatment effectiveness on a larger scale [44]. Several health data companies now collect and standardize EHR information using both structured and unstructured data [47,48].

In contrast, claims datasets primarily capture administrative and billing data, including diagnoses, procedures, costs, and payments [49,50]. While claims data offer broad coverage, they lack the clinical depth of EHRs, such as laboratory results, treatment plans, and physician notes [38].

Claims data are frequently used in RWE studies, particularly in HEOR studies [2]. Due to its broad availability and structured format, claims data allows researchers to analyze healthcare resource utilization, cost-effectiveness, and long-term outcomes on a large scale [51]. It is one of the most common data sources for assessing treatment patterns, comparing costs across interventions, and evaluating healthcare outcomes in both clinical and economic terms [52]. Claims data, including both medical and pharmacy claims, are widely used as RWD sources and may be categorized as open or closed depending on the data source and completeness of the patients’ healthcare journey [53–56]. Studies show open claims now produce statistically similar healthcare utilization estimates to closed claims after deduplication, i.e., removing duplicate records (overlapping claims or redundant patient data) to ensure data accuracy [53]. Both types of claims data serve vital roles in assessing economic effectiveness, studying rare diseases, and improving clinical trial recruitment, each offering unique advantages for research [53]. Hybrid approaches (integrating open and closed claims) are rising to balance scale and precision.

### PicnicHealth, Komodo health, and datavant

PicnicHealth and Komodo Health were the primary RWD sources used in this study, linked through Datavant’s privacy-preserving tokenization technology [57–59].

- Patients with hemophilia can enroll in the PicnicHealth platform. PicnicHealth further validates patient diagnoses through medical visits documented in their EHRs as it specializes in collating and digitizing fragmented healthcare data by retrieving medical records from various providers and consolidating them into a secure, user-friendly timeline for patients [57]. The PicnicHealth platform only contains information derived from medical records. This enables centralized access, data visualization, and patient empowerment, while also supporting medical research through anonymized longitudinal data sharing with researchers. PicnicHealth prioritizes data privacy and HIPAA compliance to ensure secure handling of patient information [12]. This ultimately results in a medically rich dataset of HB patients, albeit one comprising a small number of patients and limited cost data.
- Conversely, Komodo Health is a large claims dataset in the US. Through Komodo Health’s Healthcare Map, it aggregates and anonymizes claims, laboratory results, de-identified medical and pharmacy claims representing 330 million unique US patients, with available data from 2014 to 2021 [60,61].
- Datavant technology enables a secure linkage between these two data sources, integrating patient-level medical records from PicnicHealth with large-scale claims data from Komodo Health to generate a comprehensive, longitudinal view of each patient’s healthcare journey [18].

Combination of these two data sources through Datavant’s tokenization procedures couples PicnicHealth’s enhanced healthcare insights (comprehensive medical history curation and patient-centered data) with Komodo Health’s large-scale claims integration and advanced analytics.

## Objectives

This study had two primary objectives: 1) evaluate the accuracy of Datavant’s tokenization ability to correctly link patients found in a HB (PwHB) EHR dataset and a claims database; 2) apply tokenization to assess the completeness of two linked datasets in a subset of PwHB: EHRs from the PicnicHealth platform and insurance claims from the Komodo Health database.

## Methods and findings

This was a retrospective cohort study using longitudinal data to evaluate the number of correct patient links between two datasets, the PicnicHealth EHR dataset, and the Komodo Health claims database – from January 2015 to April 2022. The time period was selected to align with the standardized ingestion and tokenization cycles available across both data sources during that period, ensuring consistent variable capture and linkage quality. This timeframe also overlaps with the highest completeness of hemophilia-related medication and laboratory data in both datasets, supporting a balanced comparison of EHR and claims completeness.

The study cohort included 93 PwHB identified in PicnicHealth’s dataset. Key variables were those essential for understanding the diagnosis, treatment, and management of HB. These included records of hemophilia-related medications, laboratory tests routinely used in hemophilia care, and documentation of HB diagnosis. In the EHR dataset (PicnicHealth), key variables were captured through clinical notes, laboratory reports, and medication records, while in the claims dataset (Komodo Health), these were identified through Current Procedural Terminology (CPT) codes.

### Tokenization accuracy

Using Datavant, we linked tokens of 93 PwHB in the US from PicnicHealth’s dataset to tokens generated from the Komodo Health’s claims database (January 2015–April 2022) to assess the potential accuracy of tokenization (S1 Fig). All tokens were generated by Datavant using first name (phonetically via the SoundEx algorithm), last name (phonetically via the SoundEx algorithm), gender and DOB (S1 Fig). Tokens from each dataset were considered “linked” when all features of the token were the same.

In this process, duplicate tokens may be generated within a single dataset due to similarities of the individual token features, especially in patients with a common name and DOB. Of course, the tokens cannot be tested directly without accessing PII directly, which would defeat the purpose of tokenizing. Therefore, we evaluated the potential accuracy of each of the links by determining whether matches across the data sources appeared to be true PwHB using available non-PII documentation between the two datasets.

We defined a correct PwHB link from PicnicHealth to Komodo Health as a patient with documentation regarding HB diagnosis, hemophilia-related medication, and/or laboratory results routinely utilized in the management of PwHB. Additionally, we further evaluated patients who did not appear to be true PwHB in Komodo database to determine whether they had a matching ZIP code. If a patient in Komodo database was linked with one in PicnicHealth, being identified as a link, they might not actually represent the same patients. To ensure transparency and reproducibility, the analytical workflow was implemented as three structured steps: 1) token creation and linkage; 2) validation of matched patient pairs; and 3) completeness assessment of key clinical variables. The overall linkage framework is presented in S1 Table.

Linkage accuracy was evaluated through comprehensive manual review of all linked records. Linkages were classified as valid when patient-level token features aligned across datasets and the overall clinical context was consistent between data sources. Evidence of hemophilia-related diagnoses, medications, or laboratory testing in claims data was used to support completeness estimates; however, the absence of such evidence within a defined time window was not considered sufficient to classify a linkage as invalid.

In cases where linkage was technically valid, but no hemophilia-related claims were observed, these instances were still evaluated as potential claims data incompleteness. Additional contextual elements, such as ZIP code concordance, were reviewed to inform the likelihood of a valid linkage. When both disease-related claims activity and geographic indicators were absent or discordant, the certainty of a valid link increased; however, definitive confirmation of identity was not possible due to the anonymized nature of tokenized data.

### Completeness of RWD sources (EHRs/Claims)

Records linked via tokenization were further compared for completeness in medication and laboratory parameters. Variations in drug names were harmonized using standardized nomenclature, and drug eras were defined as continuous treatment periods with ≤60-day gaps. Standardization of drug names was based on generic/brand alignment following RxNorm naming conventions. For laboratory data, matching was guided by a predefined list of hemophilia-related terms prior to analysis (“coagulation,” “prothrombin,” and “thrombin”) within a 7-day window to account for reporting delays. Using the final linked dataset described above, we assessed the completeness of key data elements from two linked datasets in a subset of PwHB (Table 1). Key data elements were defined as variables essential for understanding the diagnosis, treatment, and management of HB. These included 1) medications specifically used to treat HB, (e.g., clotting factor concentrates), and 2) laboratory tests critical for diagnosing and monitoring the condition (e.g., coagulation-related tests) These elements are crucial for HB research because they enable the evaluation of treatment patterns, monitoring of disease progression, and assessment of clinical outcomes. Additionally, ZIP code and disease severity were assessed for completeness among the two datasets. R software version 4.2 was used for all analyses. Given the small sample size and descriptive intent of this methodological validation, no inferential statistical tests were performed. Analyses were limited to descriptive proportions to characterize linkage accuracy and data completeness.

**Table 1:** Metrics and coding systems for hemophilia medications and key laboratory tests.

Metric category	Specific metric	Codes
Hemophilia Medication	Monline, Rixubis, Benefix, Ixinity, Alprolix, Idelvion, AlphaNine SD	N/A – matched on name only
Key Laboratory Tests	PT	CPT: 85610, 93792, 93793, 85730; 85384; 85610; 85670, 99363–99364
	APTT	CPT: 85240; 85250; 85270; 85280; 85613; 85732, 85730, 85610
	INR	CPT: 93792, 93793, HCPS: G0248, G0249, G0250
	Coagulation Assay	CPT: 85384
	Coagulation Pathway	CPT: 85240; 85250; 85270; 85280, 85730; 85384; 85610; 85670
	Fibrinogen (coagulation factor 1)	CPT: 85385, 85384, 85385, 85635, 85670, HCPS J3590

Abbreviations: APTT: Activated Partial Thromboplastin Time; CPT: Current Procedural Terminology; HCPS: Healthcare Common Procedure Coding System; INR: International Normalized Ratio; N/A = Not Available; PT: Prothrombin Time.

The following common medications were assessed for completeness: Monline, Rixubis, Benefix, Ixinity, Alprolix, Idelvion, and AlphaNine SD (Table 1). For medications, completeness was determined by identifying whether the same product name appeared in both datasets within the same treatment era. Medications were considered matched if the same product name appeared in both datasets within the same treatment era. PicnicHealth defines an “era” as a specific period during which a particular medication is administered to a patient. In PicnicHealth, medications were identified by name only, as National Drug Codes (NDC) were not available in the dataset. In Komodo Health, medication matches were based on paid claims data referencing these names. Pharmacy claims data were extracted from the Komodo pharmacy table to identify all records containing drugs with ‘factor IX’ as an ingredient. Paid claims were flagged as ‘prophylactic’ if they met the following criteria: (1) the subsequent claim for the same drug occurred within 60 days, (2) the days_supply was greater than or equal to 2, and (3) there were at least three consecutive claims following this structure.

For more analytical context, treatment regimens involving factor IX were identified using the aforementioned logic. Drug administration dates were primarily sourced from the PicnicHealth drug_entity table, then any records without a date the cohort_drug_era table was used. In cases where no date was available in the cohort_drug_era table, the date from the drug_entity table was used. Due to the presence of multiple drug eras for the same drug and the inability to distinguish which records corresponded to which era, all possible drug era dates were considered valid. The Komodo and PicnicHealth datasets were merged based on patient token, drug name, and regimen. Komodo pharmacy claims include a date of service, which was matched to the PicnicHealth drug era if it fell between the start and end dates of the era. For drug eras lacking an end date, we assigned the start as the end date.

For laboratory data, a keyword-based matching strategy was applied. Key hemophilia-related laboratory tests were assessed: PT, APTT, International Normalized Ratio (INR), coagulation assays, and fibrinogen. The associated CPT codes for the aforementioned laboratory tests are listed in Table 1. Laboratory test completeness was assessed by matching test records within a 7-day window to account for potential delays in documentation and billing. A match for medication or laboratory documentation was defined as the presence of the same drug or laboratory test in both datasets within a 7-day window. A 7-day matching window was applied between EHR and claims datasets to account for potential delays in documentation, billing, and data entry between EHR and claims sources. The 7-day period ensures that valid matches are captured even when there are slight discrepancies in the timing of record updates, reflecting real-world administrative and clinical workflow. A 7-day window was selected to account for typical administrative delays between clinical documentation in EHRs and billing submission in claims databases. Laboratory tests and procedures are often recorded in EHRs on the date performed, while claims submission may occur several days later. A 7-day window was therefore selected to balance temporal alignment with real-world claims workflow, minimizing missed true matches due to documentation lag, while preserving clinical plausibility.

Although this approach may permit limited overmatching by associating unrelated clinical events, it was intentionally selected as a clinically and administratively appropriate window to maximize sensitivity and ensure capture of concurrent clinical activity. Shorter windows (e.g., same-day matching) risk underestimating completeness due to known administrative delays, whereas substantially longer windows could increase the likelihood of unrelated events being matched.

For more analytical context on the matching logic, we employed a keyword-based matching strategy, selecting CPT codes in Komodo associated with the term “coagulation” and aligning them with similar keywords in PicnicHealth. To enhance the scope of this matching, additional keywords including “prothrombin” and “thrombin” were incorporated. While this method does not yield precise matches due to the fact CPT is unavailable in PicnicHealth, it represents a reasonable compromise for analyses where exact code alignment is not feasible. We acknowledge that inclusion of broad keywords such as “coagulation” could potentially increase the likelihood of overmatching by capturing unrelated laboratory tests. However, this trade-off was considered acceptable to minimize false negatives and was documented to ensure transparency of the linkage process. All matches were manually reviewed to confirm clinical consistency, ensuring the methodological integrity and reproducibility of the linkage.

Completeness was then quantified as the proportion of patients with complete, partial, or no matches for each medication and key laboratory variable. For each variable, completeness was quantified as the proportion of patients with complete, partial, or no matches between datasets. The proportion metrics were defined as the following: 1) Complete Match – All medications/laboratory results in EHR found in claims, 2) Partial Match – Some medications/laboratory results matched, 3) No Match – None matched 4) Overall Completeness – Percentage of patients with certain variables (ZIP code, disease severity) present in both datasets. This stepwise, transparent matching framework allows reproducibility and provides a methodological benchmark for future tokenization validation studies in both rare and common diseases.

While this methodology does not require a specific one-to-one match, it has flexibility in identifying a match and therefore is more inclusive. We assessed the proportion of matches in Komodo Health claims of the following key variables found in PicnicHealth’s dataset: hemophilia medication and key laboratory tests utilized in the routine diagnosis and the care of hemophilia (Table 1). Additionally, we assessed the overall completeness of certain key variables (such as ZIP codes and disease severity), determining whether they were identified in each patient in both the PicnicHealth and Komodo Health datasets.

### Findings – tokenization accuracy

PicnicHealth produced tokens for 93 patients and Komodo Health produced 114 tokens. Duplicate tokens are likely produced on variation in the patient’s name found in the medical claims data. Overall, 14 patients had duplicate tokens in Komodo Health: 10 had 2 tokens, 3 had 3 tokens, and 1 had 6 tokens. PicnicHealth did not produce duplicate tokens. All 93 PicnicHealth patients appeared to link to a Komodo Health token. Komodo Health’s database contains various types of open and closed claims. Closed claims tend to contain the most comprehensive information about a patient, while an open claim contains the least, as it may be a one-off claim the patient incurred. Table 2 further describes the breakdown of links among the various claims information in Komodo Health including matches with: enrollment – both closed and open (71.0%), enrollment – closed claims only (68.8%), open claims only (4.3%) and unknown claims (29.0%).

**Table 2:** Number of matches to various types of komodo health information.

	N	Match Rate
**PwHB in PicnicHealth**	93	
**PwHB in PicnicHealth with linked token in Komodo Health**	93	100.0%
**PwHB with linked token in Komodo Health + enrollment information (both)**	66	71.0%
**PwHB with linked token in Komodo Health + enrollment information (closed claims only)**	64	68.8%
**PwHB with linked token in Komodo Health + open claims only**	4	4.3%
**PwHB with linked token in Komodo Health + unknown claims**	27	29.0%

Abbreviations: PwHB = Patients with Hemophilia B

**Fig 1:**
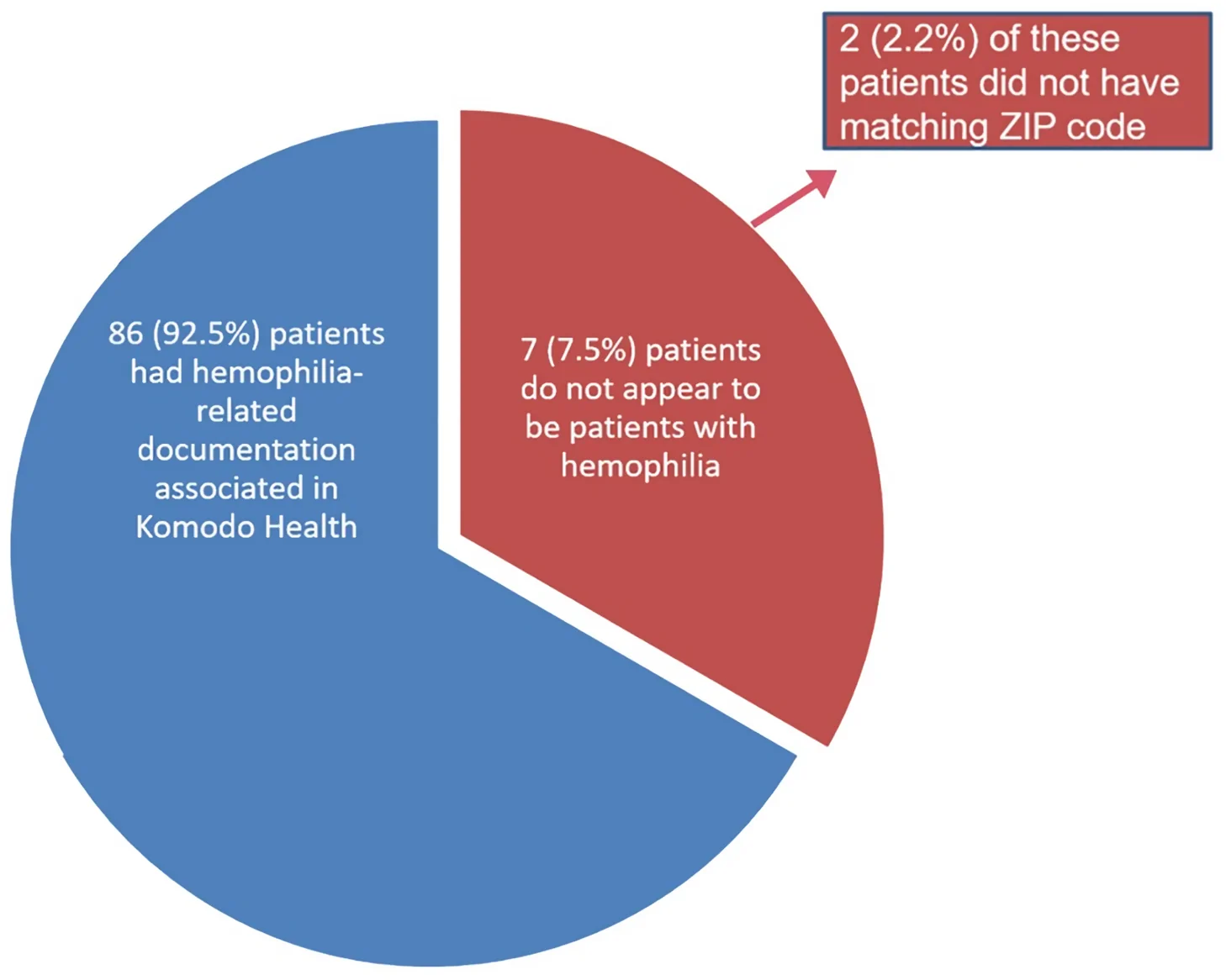
Validation of documentation of hemophilia medication, select hemophilia laboratory results or Hemophilia B diagnosis in claims database (Komodo Health, N = 93). Note: This figure is provided for visualization purposes only; slice sizes are not drawn to scale.

In the Komodo Health dataset linked with PicnicHealth patients, the majority of linkages were successful, with 93% (86 of 93) of patients demonstrating consistent documentation of hemophilia diagnosis, medication use, or laboratory data. Seven patients (7.5%) lacked explicit hemophilia-related documentation, suggesting potential non-linkage due to incomplete or non-specific data (Fig 1). Of these, two (2.2%) also had non-matching ZIP codes, further indicating possible linkage discrepancies. However, because all patient identifiers and ZIP codes are privacy-preserved and encrypted, it is not possible to confirm whether these represent true non-link or legitimate encounters occurring outside a patient’s primary residence (e.g., during travel). Based on these criteria, linkage accuracy was estimated at 93% when using hemophilia documentation alone and up to 98% when both hemophilia documentation and ZIP code concordance were considered. However, it should be noted that it is possible that hemophilia documentation was inadvertently omitted from the claims record, or the patient was traveling during the hospital visit, and the link may in fact be valid. A temporary technical error identified during initial token generation resulted in only 97% (91 of 93) of records linking successfully; this was subsequently corrected through a token refresh, restoring linkage for all 93 patients.

### Findings – Completeness of RWD sources (EHRs/Claims)

**Fig 2:**
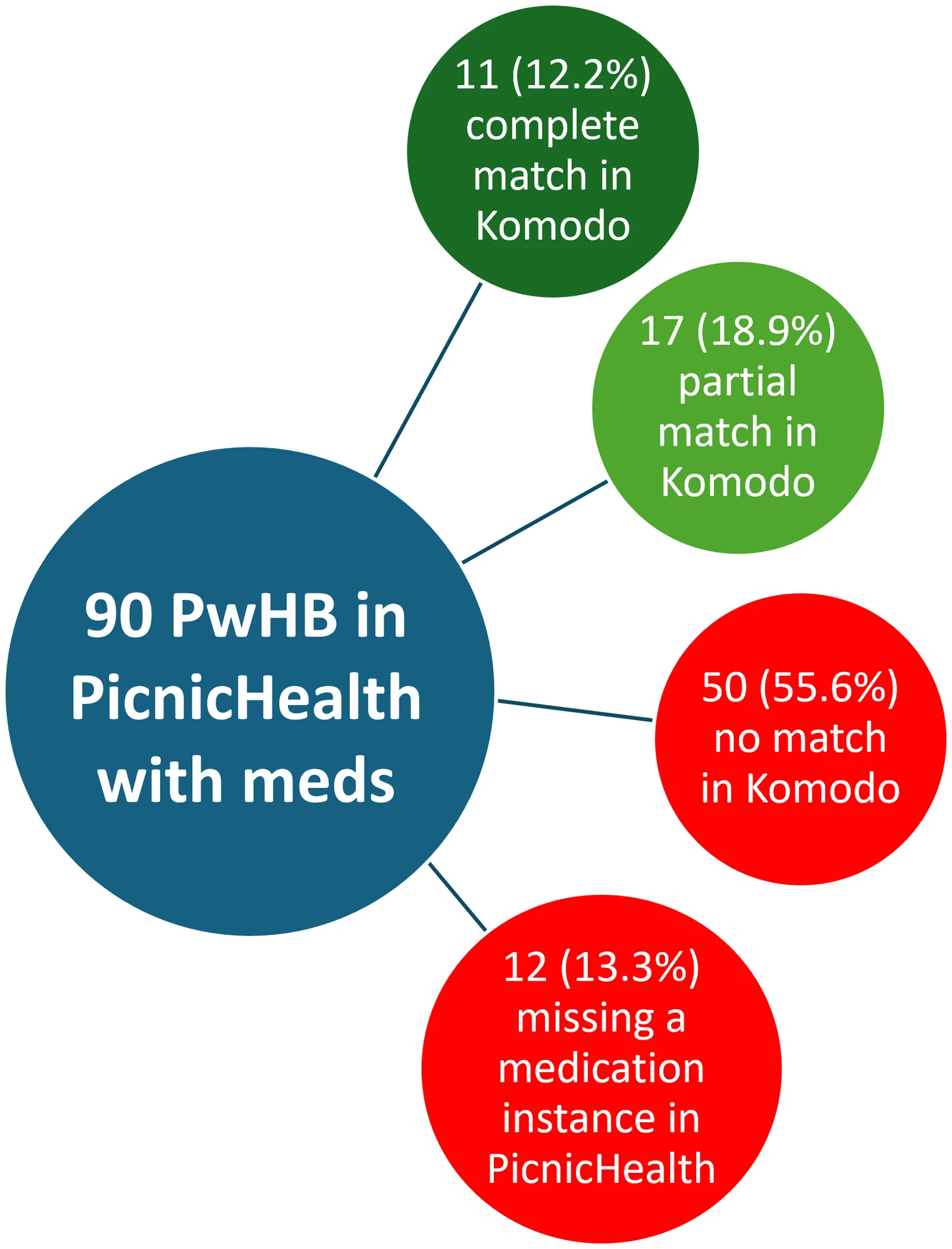
Patients in picnichealth with medication documentation (N = 90). Abbreviations: PwHB = Patients with Hemophilia B.

Of the 90 patients found in PicnicHealth with a hemophilia factor medication (Fig 2):

- 11 (12.2%) were a complete match: all medications listed in PicnicHealth were also found in Komodo
- 17 (18.9%) were a partial match: only some of the medications listed in PicnicHealth were also found in Komodo
- 50 (55.6%) had no match: none of the medications listed in PicnicHealth were found in Komodo

In terms of the completeness of medication in PicnicHealth, documentation from the two linked datasets show that 12 (13.3%) patients in PicnicHealth had missing medications that were found in Komodo Health.

**Fig 3:**
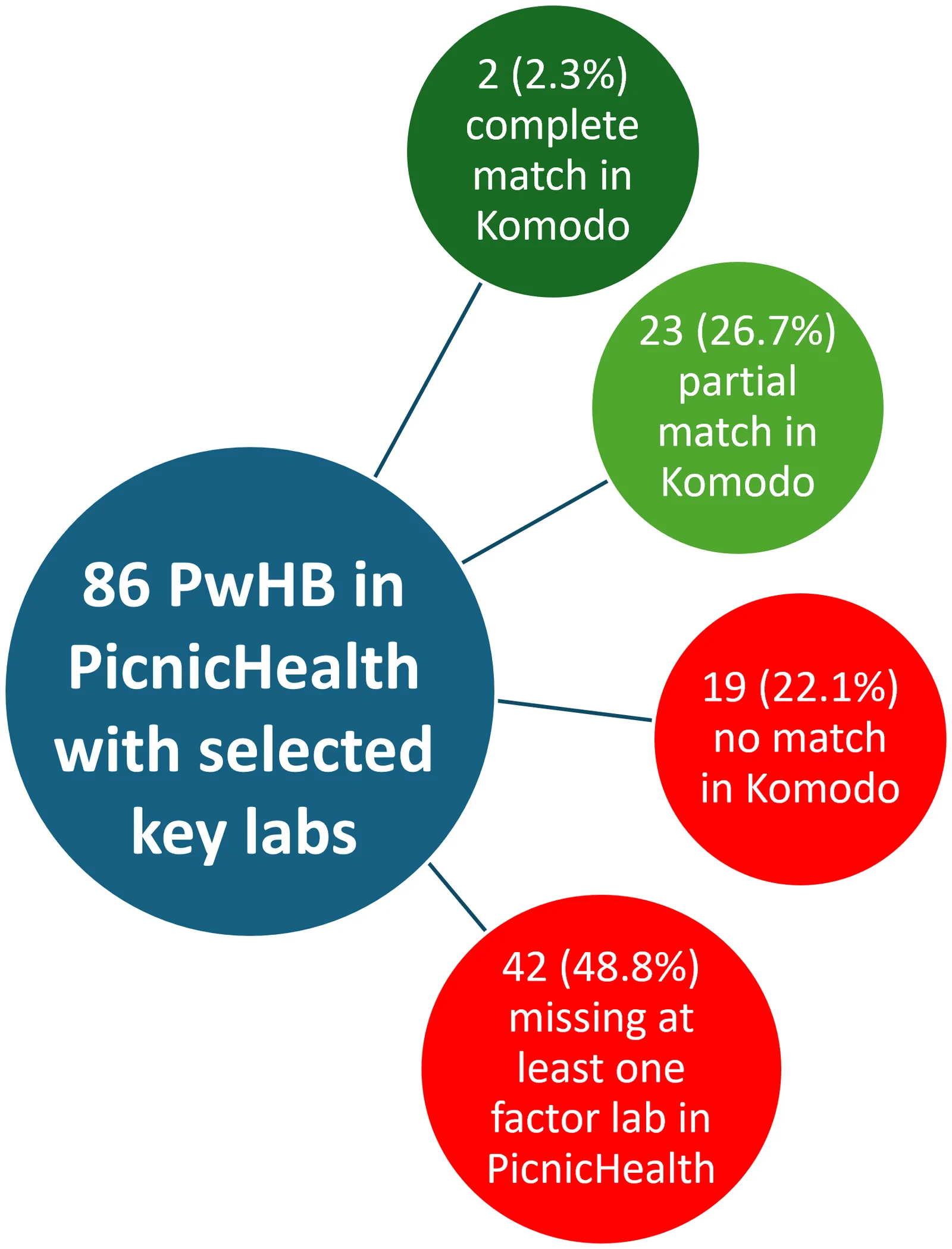
Patients in PicnicHealth with Key Laboratory Documentation (N = 86). Abbreviations: PwHB = Patients with Hemophilia B.

Of the 86 patients found in PicnicHealth with a factor laboratory documentation (Fig 3):

- 2 (2.3%) were a complete match in Komodo with all of their laboratory results listed in PicnicHealth
- 23 (26.7%) were a partial match with some of their laboratory results from PicnicHealth listed in Komodo
- 19 (22.1%) were no match – did not have any laboratory documentation listed in Komodo.

In terms of laboratory completeness in PicnicHealth, documentation from the two linked datasets show that 42 (48.8%) patients in PicnicHealth had missing laboratory results that were found in Komodo. Overall, only 12% of medication records and 2% of factor laboratory results were complete matches across datasets, underscoring substantial limitations in cross-source completeness between PicnicHealth and Komodo Health.

Additionally, we compared the overall completeness of certain variables (ZIP code, disease severity) in PicnicHealth and Komodo Health (Table 3). In PicnicHealth, the completeness of ZIP codes was 100% for all sites of healthcare encounters. A total of 90 (96.7%) patients had all factor medication, 86 (92.5%) had all select hemophilia laboratory results, and 83 (89.2%) had severity of diagnosis. In Komodo Health, the completeness of ZIP codes was 100%. A total of 41 (44.0%) had all factor medications, 68 (73.1%) had all select hemophilia laboratory results, and no patient (0%) had severity of diagnosis. While Komodo Health does not have a discrete variable indicating severity of diagnosis, it might be possible to ascertain using algorithms and information derived from factor laboratory results.

**Table 3:** Completeness of key variables from patients with Hemophilia B in EHR and claims datasets (N = 93).

	PicnicHealth	Komodo Health
**ZIP codes**	100 (100%)	100 (100%)
Factor Medication*	90 (96.7%)	41 (44.0%)
Select hemophilia labs**	86 (92.5%)	68 (73.1%)
Severity of diagnosis***	83 (89.2%)	0 (0%)

Abbreviations: APTT = Activated Partial Thromboplastin Time; CPT = Current Procedural Terminology; EHR = Electronic Health Record; INR = International Normalized Ratio; Lab = Laboratory; PT = Prothrombin Time.

*Factor medications: Monoline, Rixubis, Benefix, Ixinity, Alprolix, Idelvion, AlphaNine SD

**Factor lab codes: Prothrombin Time (CPT code: 85610, 93792, 93793, 85730; 85384; 85610; 85670, 99363–99364); APTT (CPT: 85240; 85250; 85270; 85280; 85613; 85732, 85730, 85610); INR (CPT: 93792, 93793, HCPS: G0248, G0249, G0250); Coagulation Assay (CPT 85384); Coagulation Pathway (CPT: 85240; 85250; 85270; 85280, 85730; 85384; 85610; 85670); Fibrinogen (CPT: 85385, 85384, 85385, 85635, 85670, HCPS J3590).

*** Komodo Health does not have a discrete variable indicating severity of illness, however, algorithms have been developed to label patients’ severity status in claims data. Additionally, Komodo Health includes lab data where baseline factor data to ascertain severity may be extracted.

## Conclusion

This study demonstrates that tokenization can accurately link disparate RWD, specifically EHRs and administrative claims, in a rare disease population of HB. In our data, the tokenization process achieved a high linkage accuracy rate (93% or 98%, depending on the validation variables assessed). Duplicate tokens found in this study suggest that variations of first and last name spelling/pronunciations may affect the ability to determine accuracy of matches. In larger datasets, the presence of duplicate records can complicate data analysis; if multiple entries correspond to the same individual, it can skew results and lead to erroneous conclusions [62].

When the PicnicHealth EHR data was compared to Komodo Health’s claims dataset, it appeared that PicnicHealth captured the majority of medications and laboratory results found in Komodo Health, albeit not all. However, despite the high linkage accuracy, full cross-source completeness between datasets was limited, indicating that alignment of specific medication and laboratory records remained low. Generally, greater data density was observed in the EHR dataset relative to the claims dataset for selected hemophilia-related medications and laboratory measures. This difference may reflect structural differences in data capture mechanisms across platforms, including the aggregation and curation of longitudinal EHR records and the fragmented design of claims data via disparate payers. These differences should be interpreted as variations in data architecture and intended use rather than evidence of inherent superiority of one data source over another.

This suggests that aggregated longitudinal EHR datasets like PicnicHealth, which manually extract comprehensive clinical information, may offer a more reliable and detailed source for understanding patient treatment and management in rare diseases such as HB. Komodo Health integrates diverse data, including non-claim information from various payers, but is expectedly less comprehensive in regards to medical information compared to PicnicHealth. The differences in completeness between Komodo Health and PicnicHealth are likely due to the distinct data collection methodologies used by each platform. However, claims data often have inherent limitations, such as gaps caused by payer changes or incomplete reporting, which can impact the longitudinal tracking of patient information. On the other hand, PicnicHealth collects and curates comprehensive longitudinal EHRs from patients’ healthcare providers, allowing for a more complete representation of an individual patient’s clinical history with fewer gaps.

While it is unlikely there will ever be a perfect source of information, both claims and EHR datasets have advantages for use in RWE studies and opportunities to create a near-complete patient journey. The ideal composite dataset would include both EHR data, which provide detailed information such as disease severity and laboratory result values, and claims, which include billing information on the healthcare services provided. EHRs and claims together would help identify critical gaps. Additionally, longitudinal medical record datasets, such as PicnicHealth, can be periodically linked to claims datasets, which may help to validate and identify gaps in completeness during data aggregation. Additionally, linking EHR and claims datasets via tokenization enables the creation of a more complete patient record, which can be used to verify information such as algorithms that classify treatment regimens (prophylactic vs. on demand) and disease severity. By comparing algorithm outputs against detailed clinical documentation in EHRs and healthcare utilization patterns in claims, researchers can assess and improve the accuracy of these algorithms, ensuring robust classification and generalizability across diverse data sources.

This study demonstrates that accurate patient-level tokenization between EHR and claims data is feasible even within a small rare-disease cohort such as HB. Despite a limited sample size, our findings underscore that claims data alone are insufficient to capture disease-specific clinical parameters such as severity or laboratory monitoring. Integrating patient-centric EHR data substantially enhances completeness and interpretability, particularly for rare conditions where manual record validation remains possible. These results highlight the importance of combining tokenized RWD sources to improve data accuracy, representativeness, and applicability in future rare disease research and RWE generation.

The findings underscore the broader applicability of tokenization for linking fragmented healthcare data across diverse sources, not only in rare diseases but also in more common conditions. However, the accuracy and completeness of linkage depend on the quality and stability of the underlying data elements used for token generation. Ongoing improvements in tokenization algorithms and validation protocols are essential to optimize linkage reliability, especially in larger and more heterogeneous populations.

In conclusion, tokenization offers a scalable and privacy-compliant solution for integrating RWD, facilitating longitudinal research, validation and enhancing the validity of RWE studies. The absence of extensive research applying tokenization in rare diseases points to a significant gap. Future studies are needed to explore how tokenization can be used to link scarce and dispersed data sources in rare disease populations, where capturing adequately-sized cohorts remains challenging.

Future research should also focus on validating this study’s findings in larger cohorts and exploring strategies to address data completeness and technical challenges inherent in token-based linkage.

## Limitations

Linkage performance may be influenced by the availability and completeness of identifying variables across datasets, as well as technical implementation factors during token generation, which may introduce minor inaccuracies or missed links.

In our data, tokenization achieved a high accuracy rate, however, technical errors and incomplete PII may have affected matching precision. Due to privacy regulations, identifying information underlying the tokens was not accessible for direct verification of linkages. In addition, duplicate tokens arising from variations in name spelling or pronunciation may have introduced minor inaccuracies. Consequently, tokenization in this study was assessed indirectly through consistency in clinical documentation rather than through direct identity verification. Given the small sample size and the descriptive nature of this methodological validation, inferential statistical testing was not conducted; instead, analyses were limited to descriptive proportions to characterize linkage accuracy and data completeness.

In our study, it appears that the proportion of valid tokenized links was high (98% based on hemophilia-related documentation and 93% when including ZIP code). Therefore, patients that may not have a valid link could influence the results of the completeness assessment of variables found in PicnicHealth and Komodo Health. Additionally, differences in database structure may have influenced the matching logic used to link medication and factor laboratory data, potentially affecting estimates of concordance between these measures. For example, PicnicHealth does not contain NDC codes, so matching was based on medication name only. Additionally, selected keywords in Komodo Health (e.g., “coagulation”) were considered relevant matches to laboratory CPT code names found in PicnicHealth. These factors highlight the importance of distinguishing between linkage performance and underlying data completeness when interpreting observed discordances.

The use of a 7-day matching window may have introduced a modest risk of overmatching, whereby unrelated laboratory events occurring within the window could be misclassified as concordant across datasets. Although the window was selected to reflect real-world claims and documentation practices, alternative temporal definitions (e.g., same-day or shorter intervals) may yield different completeness estimates. Future studies in larger cohorts could formally evaluate the impact of varying window definitions on matching performance. An aggregated EHR data source such as PicnicHealth may vary in completeness compared with EHR systems maintained within structured health systems. Accordingly, the findings may not be fully representative of all individuals with hemophilia B or the level of data completeness observed in health system–based EHR datasets. The results are therefore most applicable to patient-mediated EHR aggregation contexts.

Finally, the study was conducted on a small cohort of 93 patients and findings should be interpreted within this specific dataset context. Future research with larger and more diverse patient populations is needed to validate these results and further assess the accuracy and completeness of tokenization across different datasets [63,64].

## Supporting information

S1 Fig Datavant technology. Data were de-identified by removing PHI and replacing it with encrypted keys.
(DOCX)

S1 Table Token designs. Datavant technology combines different PII elements to create unique, irreversible tokens.
(DOCX)
