# Supplementary material for: Evaluation of the completeness of a medical record dataset linked to administrative claims through tokenization in patients with Hemophilia B
Source: PLoS One. 2026 Sep 8;21(9):e0355051. doi: 10.1371/journal.pone.0355051 (PMC13552784; doi:10.1371/journal.pone.0355051)
Supplement: Supplementary file 1 [file pone.0355051.s001.docx]

**Figure S1. Datavant Technology**

Data was de-identified by removing PHI and replacing it with encrypted keys

**Configured template with rules**

***Create encrypted key (“token”)
from name, DOB and gender***

**Input data**

**Output data**

AA001

Abbreviations: DOB: Date of birth; PHI: Patient’s Health Information; Identifiable Information

**Table S1. Token Designs**

Datavant technology combines different PII elements to create unique, irreversible tokens

| **Name** | **Token Elements** |
| --- | --- |
| **Token 01** | Last Name + 1st Initial of First Name + Gender + DOB |
| **Token 02** | Last Name (SoundEx) + First Name (SoundEx) + Gender + DOB |
| **Token 03** | Last Name + First Name + DOB + ZIP3 |
| **Token 04** | Last Name + First Name + Gender + DOB |
| **Token 05** | SSN + DOB + Gender |
| **Token 16** | SSN + First Name |
| **Token 17** | Last Name + First Name + Address + ZIP5 |
| **Token 29** | First Name + Email |
| **Token 30** | First Name + Cell Phone |

Abbreviations: DOB: Date of birth; PII: Personally Identifiable Information; SSN: Social Security Number
