# Supplementary material for: Evaluation of the completeness of a medical record dataset linked to administrative claims through tokenization in patients with Hemophilia B
Source: PLoS One. 2026 Sep 8;21(9):e0355051. doi: 10.1371/journal.pone.0355051 (PMC13552784; doi:10.1371/journal.pone.0355051)
Supplement: Supplementary file 3 [file pone.0355051.s003.docx]

Manuscript Title:

**Evaluation of the Completeness of a Medical Record Dataset Linked to Administrative Claims through Tokenization in Patients with Hemophilia B**

Dear Editors and Reviewers,

We thank the reviewers for their thoughtful and constructive feedback. Below is our point-by-point response, with corresponding revisions incorporated into the manuscript.

1. Reframing the Core Contribution and Novelty

**Reviewer Comment: The manuscript does not sufficiently articulate its unique contribution.**

Response:
We agree and have revised the Introduction and Methods to emphasize the methodological advantage of using Hemophilia B (HB) as an ideal test case and how the results can be generalized. HB’s rarity enables manual review of all records, allowing for a direct and accurate assessment of tokenization accuracy. This approach is not feasible in large disease populations such as cancer or diabetes. This differentiates our study from broader works (e.g., Tyagi et al.) and highlights its unique value for rare disease research. We also clarify how these findings inform best practices for integrating claims and EHR data which is needed to create large enough sample sizes that contain rich data in rare disease RWE studies.

2. Methodological Transparency and Reproducibility

**Reviewer Comment: Insufficient detail on data processing and matching methodology.**

Response:
We have expanded the Methods section to include a detailed, step-by-step description of the matching algorithm:
- Drug Name Handling: Variations (generic vs. brand) normalized using RxNorm mappings
- Medication Era Definition: Continuous treatment with ≤60-day gaps

-Dataset Tables: Table names were included to better understand data collation and analyzation.
- Lab Test Matching: Keywords included “coagulation,” “prothrombin,” and “thrombin,” selected for clinical relevance to HB
- 7-Day Window Justification: Chosen to balance completeness and specificity, minimizing missed matches while acknowledging potential overmatching
The acknowledgment of overmatching has been moved from Limitations to Methods, with justification provided.

3. Lack of Statistical Depth

**Reviewer Comment: Analysis limited to descriptive statistics.**

Response:
We do not want to present statistics that may mislead readers in the intent of our study. We included the following sentence to clarify our intent. “Given the small sample size and the descriptive intent of this methodological validation, inferential statistical tests were not performed; instead, analyses were limited to descriptive proportions to characterize linkage accuracy and data completeness.”

4. Justification and Discussion

**Reviewer Comment: Rationale for dataset timeframe and lack of limitations in abstract/conclusion.**

Response:
The retrospective period (Jan 2015–Apr 2022) was selected as claims data typically have a lag for access to full dataset. More recent data were not available at the time of analysis. We have revised the Abstract to include key limitations (small cohort size) and updated the Conclusion to assert practical lessons:
“The study’s findings are limited by the small sample size and retrospective design, which may affect generalizability, however this study provides foundational description. Nonetheless, the study also provides valuable methodological insights into tokenization accuracy and the relative strengths of EHR and claims data for RWE generation”

5. Financial Disclosure

**Reviewer Comment: Discrepancy between acknowledgments and disclosure statement.**

Response:
We have corrected the Financial Disclosure to state:
“This research was conducted by employees of Pfizer Inc. using data licensed from PicnicHealth and Komodo Health. No external or dedicated funding was received for this work. The research was conducted by full-time employees of Pfizer Inc. as part of their regular responsibilities.”
This aligns with journal guidelines and resolves the previous inconsistency.

Summary of Major Revisions

- Introduction and Methods revised to highlight novelty and methodological advantage.

- Detailed algorithm description added for reproducibility.

- Statistical analyses are not needed nor necessary for this type of descriptive study.

- Abstract and Conclusion updated to include limitations and practical implications.

- Financial Disclosure corrected for clarity and compliance.

We appreciate the reviewers’ insights, which have significantly strengthened our manuscript. Please let us know if further clarification is needed.

Sincerely,
Diana Stan
On behalf of all authors
