# Supplementary material for: Evaluation of the completeness of a medical record dataset linked to administrative claims through tokenization in patients with Hemophilia B
Source: PLoS One. 2026 Sep 8;21(9):e0355051. doi: 10.1371/journal.pone.0355051 (PMC13552784; doi:10.1371/journal.pone.0355051)
Supplement: Supplementary file 5 [file pone.0355051.s005.docx]

**Evaluation of the Completeness of a Medical Record Dataset Linked to Administrative Claims through Tokenization in Patients with Hemophilia B**

Dear Editors and Reviewers,

We thank the Editor and Reviewer 2 for the additional thoughtful comments and suggestions. We have carefully revised the manuscript and provide our point-by-point responses below.

### **Comment 1:**

*‘A major dispute is whether 95% confidence interval calculation should be included. I agree that this is unnecessary and may be misleading for the actual observations, instead of estimates. Please add “in our data” following “achieved a high linkage accuracy rate (93–98%)” in line 373.’*

### **Response:**

We thank the Editor for this clarification and agree that confidence intervals are unnecessary in the context of this descriptive methodological evaluation. As suggested, we revised the conclusion to clarify that these values represent observed findings within our study sample rather than inferential population estimates. The sentence was revised to:

“In our data, the tokenization process demonstrated high linkage accuracy (93% or 98%, depending on the validation variables assessed).” This revision also clarifies that the reported values reflect different validation approaches rather than a statistical interval.

### **Comment 2:**

*‘Regarding the “7-day matching window” comment, can you compare the matching rates at 0, 3, 7 and 15 days as suggested by the reviewer? If you have good reasons to skip this analysis, such as feasibility issues, please elaborate.’*

### **Response:**

We appreciate this suggestion and agree that evaluation of alternative matching windows would be informative. However, conducting additional sensitivity analyses was not feasible within the current revision process because the analyses were originally conducted within a third-party hosted environment that is no longer accessible following completion of the project collaboration.

To address this concern, we further clarified in the Limitations section that alternative temporal definitions may yield different completeness estimates and that future studies in larger cohorts should formally evaluate the impact of varying window definitions on matching performance.

### **Comment 3:**

*‘Regarding the reviewer’s concern that PicnicHealth is a patient self-registration platform, does the patient have a role beyond registration? The manuscript mentioned in passing that healthcare providers may also enter data into this EHR. It may be helpful to specify the roles of patients and healthcare providers in PicnicHealth data accumulation.’*

### **Response:**

We appreciate the opportunity to clarify this point and revised the manuscript accordingly. Patients may enroll into the PicnicHealth platform; however, the EHR data itself is passively collected from existing medical records by the PicnicHealth abstractors. Neither patients nor healthcare providers directly enter clinical information into the PicnicHealth platform for purposes of this study. More information about the PicnicHealth platform and the abstraction process can be found here: Picnichealth.com

We revised the manuscript to clarify that PicnicHealth validates diagnoses using medical records and that the platform contains data derived from the underlying medical records obtained from healthcare providers.

### **Comment 4:**

*‘Please respond to the comment that “Overemphasizing the ‘98%’ figure in the conclusion is an overstatement that exceeds the limitations of the study design”.’*

### **Response:**

We appreciate this comment and revised the conclusion to avoid overstating the findings. Specifically, we clarified that the linkage accuracy values reflect observations within our study sample and revised the wording to present the findings in a more descriptive and methodologic manner. This wording better contextualizes the findings within the limitations of the study design.

### **Additional Revision:**

In response to the Editor’s suggestion, we also consolidated the Limitations section to improve readability while retaining the major methodological considerations and contextual points raised during peer review.

We thank the Editor and Reviewer for their valuable feedback, which has helped improve the clarity and methodological transparency of the manuscript.

Sincerely,
Diana Stan
On behalf of all authors
