# Supplementary material for: Evaluation of the completeness of a medical record dataset linked to administrative claims through tokenization in patients with Hemophilia B
Source: PLoS One. 2026 Sep 8;21(9):e0355051. doi: 10.1371/journal.pone.0355051 (PMC13552784; doi:10.1371/journal.pone.0355051)
Supplement: Peer review history

# Peer review: Decision Letter 0

**DOI:** 10.1371/journal.pone.0355051.r001

**Authors:** Yosi Kristian

29 Sep 2025

Dear Dr. Stan,

Thank you for submitting your manuscript to PLOS ONE. After careful consideration, we feel that it has merit but does not fully meet PLOS ONE’s publication criteria as it currently stands. Therefore, we invite you to submit a revised version of the manuscript that addresses the points raised during the review process.

Of course. Here are a few options for a concise email body, ranging from a brief summary to a more detailed one.

<h3>**Option 1: Very Concise Summary (Ideal if the full letter is attached)** </h3>

**Subject:**  Decision on your PLOS ONE manuscript PONE-D-25-21079

Dear Dr. Stan,

Thank you for your submission to PLOS ONE. We have completed the review of your manuscript, "Evaluation of the Completeness of a Medical Record Dataset Linked to Administrative Claims through Tokenization in Patients with Hemophilia B."

After careful consideration of the feedback from our expert reviewers, we have determined that your manuscript requires **Major Revision**  before it can be reconsidered for publication.

The required revisions fall into five main categories:

- Reframing the core contribution and novelty of using a rare disease cohort.
- Improving methodological transparency and reproducibility.
- Incorporating more rigorous statistical analysis.
- Strengthening the justification of study parameters and discussion of limitations.
- Providing a critical correction to the financial disclosure statement.

Please see the decision letter below for a detailed breakdown of the required changes.

Please submit your revised manuscript by Nov 13 2025 11:59PM. If you will need more time than this to complete your revisions, please reply to this message or contact the journal office at plosone@plos.org. When you're ready to submit your revision, log on to https://www.editorialmanager.com/pone/ and select the 'Submissions Needing Revision' folder to locate your manuscript file.

- A rebuttal letter that responds to each point raised by the academic editor and reviewer(s). You should upload this letter as a separate file labeled 'Response to Reviewers'.
- A marked-up copy of your manuscript that highlights changes made to the original version. You should upload this as a separate file labeled 'Revised Manuscript with Track Changes'.
- An unmarked version of your revised paper without tracked changes. You should upload this as a separate file labeled 'Manuscript'.

If you would like to make changes to your financial disclosure, please include your updated statement in your cover letter. Guidelines for resubmitting your figure files are available below the reviewer comments at the end of this letter.

If applicable, we recommend that you deposit your laboratory protocols in protocols.io to enhance the reproducibility of your results. Protocols.io assigns your protocol its own identifier (DOI) so that it can be cited independently in the future. For instructions see: https://journals.plos.org/plosone/s/submission-guidelines#loc-laboratory-protocols. Additionally, PLOS ONE offers an option for publishing peer-reviewed Lab Protocol articles, which describe protocols hosted on protocols.io. Read more information on sharing protocols at https://plos.org/protocols?utm_medium=editorial-email&utm_source=authorletters&utm_campaign=protocols.

We look forward to receiving your revised manuscript.

Kind regards,

Yosi Kristian

Academic Editor

PLOS ONE

Journal requirements:

When submitting your revision, we need you to address these additional requirements.

1. Please ensure that your manuscript meets PLOS ONE's style requirements, including those for file naming. The PLOS ONE style templates can be found at

https://journals.plos.org/plosone/s/file?id=wjVg/PLOSOne_formatting_sample_main_body.pdf and

https://journals.plos.org/plosone/s/file?id=ba62/PLOSOne_formatting_sample_title_authors_affiliations.pdf

2. We note that there is identifying data in the Supporting Information file <Appendix.docx>. Due to the inclusion of these potentially identifying data, we have removed this file from your file inventory. Prior to sharing human research participant data, authors should consult with an ethics committee to ensure data are shared in accordance with participant consent and all applicable local laws.

Data sharing should never compromise participant privacy. It is therefore not appropriate to publicly share personally identifiable data on human research participants. The following are examples of data that should not be shared:

-Name, initials, physical address

-Ages more specific than whole numbers

-Internet protocol (IP) address

-Specific dates (birth dates, death dates, examination dates, etc.)

-Contact information such as phone number or email address

-Location data

-ID numbers that seem specific (long numbers, include initials, titled “Hospital ID”) rather than random (small numbers in numerical order)

Data that are not directly identifying may also be inappropriate to share, as in combination they can become identifying. For example, data collected from a small group of participants, vulnerable populations, or private groups should not be shared if they involve indirect identifiers (such as sex, ethnicity, location, etc.) that may risk the identification of study participants.

Additional guidance on preparing raw data for publication can be found in our Data Policy (https://journals.plos.org/plosone/s/data-availability#loc-human-research-participant-data-and-other-sensitive-data) and in the following article: http://www.bmj.com/content/340/bmj.c181.long.

Please remove or anonymize all personal information, ensure that the data shared are in accordance with participant consent, and re-upload a fully anonymized data set. Please note that spreadsheet columns with personal information must be removed and not hidden as all hidden columns will appear in the published file.

If the reviewer comments include a recommendation to cite specific previously published works, please review and evaluate these publications to determine whether they are relevant and should be cited. There is no requirement to cite these works unless the editor has indicated otherwise.

Additional Editor Comment:

The manuscript has now been evaluated by several expert reviewers. We have received their feedback, and after careful consideration, we find that while the topic is timely and holds significant interest, substantial revisions are required before it can be accepted for publication. Therefore, we are recommending a decision of Major Revision. The reviewers acknowledged the importance of evaluating data linkage methods in real-world evidence (RWE) studies, particularly for rare diseases. However, they raised several critical concerns that must be addressed. I have synthesized the key points from their reports below, which should guide your revisions.

1. Reframing the Core Contribution and Novelty. A primary concern is that the manuscript does not sufficiently articulate its unique contribution. The reviewers noted that while you state Hemophilia B (HB) is an "ideal use case," you fail to emphasize why this is a methodological advantage. You must clearly explain that the low incidence of this rare disease makes manual record review feasible, allowing for a more direct and accurate assessment of tokenization accuracy than is possible in large cohorts of common diseases (e.g., cancer, diabetes). This key advantage should be a central argument in your Introduction and Methods sections. Please differentiate your work from broader studies (e.g., Tyagi et al.), clarifying how your findings specifically add value to the rare disease research community beyond what is already known about tokenization in larger, more diverse populations.

2. Methodological Transparency and Reproducibility. The reviewers unanimously found the description of the data processing and matching methodology to be insufficient for ensuring reproducibility. In the Methods section, please provide a detailed, step-by-step description of your matching algorithm. This must include: How variations in drug names (e.g., generic vs. brand) were handled, A precise definition of the "era" used for matching medication records, The specific "keywords" used for matching lab tests and the rationale for their selection,

A justification for the 7-day window for lab test matching. You acknowledge potential overmatching due to the use of keywords like "coagulation." This self-criticism should be moved from the Limitations to the Methods section, accompanied by a justification for why this approach was chosen despite its potential drawbacks.

3. Lack of Statistical Depth. The analysis is currently limited to descriptive statistics. To strengthen your conclusions, a more rigorous statistical approach is necessary. Please calculate and report confidence intervals for your tokenization accuracy estimate (e.g., the 97-98% rate).When comparing the completeness of the PicnicHealth and Komodo Health datasets (Table 3), you must perform appropriate statistical tests to determine if the observed differences are statistically significant. Simply presenting percentages is not sufficient.

4. Justification and Discussion. Several aspects of the study design and its implications require further clarification. Please provide a clear rationale for limiting the retrospective dataset to the period of January 2015 - April 2022. The abstract must be revised to include a brief statement on the study's key limitations (e.g., small cohort size). The Conclusion should be rewritten to be more assertive and to clearly state the practical lessons learned. For instance, you could emphasize that for rare diseases, claims data alone is insufficient and must be integrated with detailed, patient-centric EHR data to capture critical information like disease severity.

5. Critical Administrative Correction: Financial Disclosure. A significant conflict was identified between your disclosure statement and the information in the submission form. You state in the acknowledgments that "This study was supported by Pfizer Inc." and the author affiliations are with Pfizer. However, the Financial Disclosure section notes that "The author(s) received no specific funding for this work." This is a serious discrepancy. You must provide a clear, accurate, and unambiguous disclosure statement that clarifies the exact nature of Pfizer's support (e.g., salaries for the authors, paid subscriptions to databases, direct funding) as per the journal's submission guidelines.

We believe that addressing these points will significantly strengthen your manuscript. Please provide a point-by-point response to these comments along with your revised manuscript. Note that the revised version may be sent back to the reviewers for further evaluation.

[Note: HTML markup is below. Please do not edit.]

Reviewers' comments:

Reviewer's Responses to Questions

**Comments to the Author**

1. Is the manuscript technically sound, and do the data support the conclusions?

Reviewer #1: Partly

Reviewer #2: Yes

**********

2. Has the statistical analysis been performed appropriately and rigorously?

Reviewer #1: N/A

Reviewer #2: No

**********

3. Have the authors made all data underlying the findings in their manuscript fully available?

The PLOS Data policy

Reviewer #1: Yes

Reviewer #2: Yes

**********

4. Is the manuscript presented in an intelligible fashion and written in standard English?

Reviewer #1: Yes

Reviewer #2: Yes

**********

Reviewer #1: Thank you for your submission. Interesting approach from case selection to your attempts at demonstrating the potential of tokenization in healthcare.

Language is appropriate.

References appear to be somewhat relevant but will need edits. It seems like there are some duplicates in reference no. 11 and 14 as well as reference no. 10 and 19.

1. Considering the rarity of this inherited disease (population), perhaps the high tokenization accuracy might not be unexpected? A moot point perhaps. Anyway, the authors recommended a larger and more diverse patient population for future research, which is reasonable. However, one could argue that this is somewhat attempted recently by Tyagi et al alongside suggestions for enhancing accuracy (also referenced in your manuscript as Reference No. 11 and 14). Inadvertently undermining the purpose of your research on tokenization in rare disease population. It would be good to better understand how your research adds value to the rare disease population and what your recommendations are for future research in rare disease populations. Would the authors care to comment?

2. Why was the retrospective dataset limited to Jan 2015 - Apr 2022? Was access to more recent dataset not available? Or was this a conscious decision to exclude more recent dataset? One is left to wonder if your conclusion would remain the same with the inclusion of more recent dataset.

3. Would the authors be able to elaborate on the disclosure statement that Pfizer Inc supported the study but in the response on Financial Disclosure, the author(s) have noted that no specific funding was received for this work. It appears that the authors are using email addresses hosted by Pfizer. Would the authors also comment if access to the databases/software were via public general access or paid subscriptions by Pfizer? An accurate disclosure must be included (refer to submission guidelines).

Reviewer #2: Overall

The authors' research addresses a timely and important topic: evaluating the accuracy of data linkages using tokenization technology and the completeness of datasets for the rare disease hemophilia B (HB). However, the paper fails to fully explain why this research is important and what benefits it offers that are not available with other datasets.

(1.1) Lack of discussion of the importance and usefulness of this research in RWD/RWE.

> Background - Hemophilia B: "HB is an ideal use case for evaluating various RWD sources, being a rare condition that is less susceptible to erroneous linkages via tokenization." (p.5)

> Overall Introduction/Background: While much space is devoted to general explanations of RWD/RWE, tokenization, EHRs, and claims data, there is little discussion of why these are particularly important in the context of the rare disease hemophilia B.

The most significant contribution of this study lies in its use of a unique dataset, the rare disease hemophilia B, to examine the issues of data linkage and data completeness, which are fundamental to RWD/RWE research. However, current literature does not adequately address the importance and usefulness of these approaches for rare diseases.

Many RWD/RWE studies and tokenization accuracy studies have focused on diseases with large patient populations, such as cancer and diabetes. The sheer volume of data for these diseases makes it impossible to scrutinize individual records, making linkage error assessment difficult. This study has a clear methodological advantage in that it focuses on rare diseases, where manual review of all records is relatively easy, allowing for a more direct and accurate assessment of tokenization accuracy. This point should be emphasized in the introduction.

The discovery that severity information is missing from claims data (Table 3) is an important finding that demonstrates the limitations of RWD in rare disease research. The conclusion should clearly state the practical lessons learned from this study: "In RWE studies of rare diseases, claims data alone is insufficient; it is essential to integrate detailed clinical information (especially severity) obtained from patient-centric EHR data such as PicnicHealth."

The usefulness of this study lies precisely in the fact that it "performed validation not possible with other common disease datasets." The `Methods` section should emphasize the unique value of this dataset, stating that "its low incidence of coincidental overlaps and the ability to facilitate manual review make it an ideal test case for assessing the true accuracy of tokenization."

(2) Lack of Transparency in Data Utilization and Preprocessing

> Methods and Findings - Completeness of RWD Sources (EHRs/Claims): "In PicnicHealth, key data elements were identified through patient-reported data, medical records, and medication names... as well as lab tests using keywords and CPT codes. In Komodo Health, medications were defined using paid claims data, and lab tests were captured through claims-based CPT codes." (p.11, lines 228-232)

> Table 1. Metrics and Coding Systems...: A list of CPT codes and medication names is presented.

The explanation of the data utilization and preprocessing process is insufficient to ensure reproducibility.

You state that "a match was defined as a match between a PicnicHealth drug name found in Komodo Health claims data during the same period," but the specific algorithm and process are unclear. How did you handle variations in drug name spellings (e.g., generic and brand names)? What is the definition of "period (era)"? What exactly does "keywords" refer to in the context of lab test matching? What is the rationale for setting a 7-day window?

Self-criticism in the Limitations section: The authors themselves admit in the `Limitations` section that "drug names were matched by name only (NDC codes were not used)" and "certain keywords, such as 'coagulation', were considered matches, which may have resulted in overmatches." While this is an honest statement, shouldn't they have provided a detailed explanation in the Methodology section to justify their choices?

(3) Lack of Discussion of the Validity of the Analytical Method

The entire paper contains almost no description of statistical analysis or models.

This study focuses solely on descriptive statistics (calculating percentages) and does not include any analysis using statistical models or discussion of the validity of the method. For example, the authors conclude that the tokenization accuracy was 97-98%, but do not calculate a confidence interval for this estimate. Furthermore, while the completeness of the two data sources is compared, the authors do not test whether the difference is statistically significant.

(4) Description of Limitations in the Abstract and Conclusions

The abstract contains no description of the study's limitations. The conclusions mention future research directions, but do not adequately discuss the limitations of the study itself.

A brief description of the study's major limitations should be added, along the lines of, "However, this study was based on a small cohort of 93 participants, and caution is advised in generalizing the results."

**********

what does this mean?). If published, this will include your full peer review and any attached files.

If you choose “no”, your identity will remain anonymous but your review may still be made public.

**Do you want your identity to be public for this peer review?**  For information about this choice, including consent withdrawal, please see our Privacy Policy

Reviewer #1: No

Reviewer #2: **Yes:**  Yasuko Kawahata

**********

[NOTE: If reviewer comments were submitted as an attachment file, they will be attached to this email and accessible via the submission site. Please log into your account, locate the manuscript record, and check for the action link "View Attachments". If this link does not appear, there are no attachment files.]

While revising your submission, please upload your figure files to the Preflight Analysis and Conversion Engine (PACE) digital diagnostic tool, https://pacev2.apexcovantage.com/. PACE helps ensure that figures meet PLOS requirements. To use PACE, you must first register as a user. Registration is free. Then, login and navigate to the UPLOAD tab, where you will find detailed instructions on how to use the tool. If you encounter any issues or have any questions when using PACE, please email PLOS at figures@plos.org. Please note that Supporting Information files do not need this step.

# Author comment: Author response to Decision Letter 1

**DOI:** 10.1371/journal.pone.0355051.r002

20 Nov 2025

Dear Editors and Reviewers,

We thank the reviewers for their thoughtful and constructive feedback. Below is our point-by-point response, with corresponding revisions incorporated into the manuscript.

1. Reframing the Core Contribution and Novelty

Reviewer Comment: The manuscript does not sufficiently articulate its unique contribution.

Response:

We agree and have revised the Introduction and Methods to emphasize the methodological advantage of using Hemophilia B (HB) as an ideal test case and how the results can be generalized. HB’s rarity enables manual review of all records, allowing for a direct and accurate assessment of tokenization accuracy. This approach is not feasible in large disease populations such as cancer or diabetes. This differentiates our study from broader works (e.g., Tyagi et al.) and highlights its unique value for rare disease research. We also clarify how these findings inform best practices for integrating claims and EHR data which is needed to create large enough sample sizes that contain rich data in rare disease RWE studies.

2. Methodological Transparency and Reproducibility

Reviewer Comment: Insufficient detail on data processing and matching methodology.

Response:

We have expanded the Methods section to include a detailed, step-by-step description of the matching algorithm:

- Drug Name Handling: Variations (generic vs. brand) normalized using RxNorm mappings

- Medication Era Definition: Continuous treatment with ≤60-day gaps

-Dataset Tables: Table names were included to better understand data collation and analyzation.

- Lab Test Matching: Keywords included “coagulation,” “prothrombin,” and “thrombin,” selected for clinical relevance to HB

- 7-Day Window Justification: Chosen to balance completeness and specificity, minimizing missed matches while acknowledging potential overmatching

The acknowledgment of overmatching has been moved from Limitations to Methods, with justification provided.

3. Lack of Statistical Depth

Reviewer Comment: Analysis limited to descriptive statistics.

Response:

We do not want to present statistics that may mislead readers in the intent of our study. We included the following sentence to clarify our intent. “Given the small sample size and the descriptive intent of this methodological validation, inferential statistical tests were not performed; instead, analyses were limited to descriptive proportions to characterize linkage accuracy and data completeness.”

4. Justification and Discussion

Reviewer Comment: Rationale for dataset timeframe and lack of limitations in abstract/conclusion.

Response:

The retrospective period (Jan 2015–Apr 2022) was selected as claims data typically have a lag for access to full dataset. More recent data were not available at the time of analysis. We have revised the Abstract to include key limitations (small cohort size) and updated the Conclusion to assert practical lessons:

“The study’s findings are limited by the small sample size and retrospective design, which may affect generalizability, however this study provides foundational description. Nonetheless, the study also provides valuable methodological insights into tokenization accuracy and the relative strengths of EHR and claims data for RWE generation”

5. Financial Disclosure

Reviewer Comment: Discrepancy between acknowledgments and disclosure statement.

Response:

We have corrected the Financial Disclosure to state:

“This research was conducted by employees of Pfizer Inc. using data licensed from PicnicHealth and Komodo Health. No external or dedicated funding was received for this work. The research was conducted by full-time employees of Pfizer Inc. as part of their regular responsibilities.”

This aligns with journal guidelines and resolves the previous inconsistency.

Summary of Major Revisions

- Introduction and Methods revised to highlight novelty and methodological advantage.

- Detailed algorithm description added for reproducibility.

- Statistical analyses are not needed nor necessary for this type of descriptive study.

- Abstract and Conclusion updated to include limitations and practical implications.

- Financial Disclosure corrected for clarity and compliance.

We appreciate the reviewers’ insights, which have significantly strengthened our manuscript. Please let us know if further clarification is needed.

Sincerely,

Diana Stan

On behalf of all authors

Attachment Submitted filename: Rebuttal Letter_PLoSOne.docx

# Peer review: Decision Letter 1

**DOI:** 10.1371/journal.pone.0355051.r003

**Authors:** Bin Zhang

15 Jan 2026

Dear Dr. Stan,

Thank you for submitting your revised manuscript to PLOS ONE. Reviewer 2 had additional comments that need to be properly addressed before this manuscript can be accepted for publication. Therefore, we invite you to submit a revised version of the manuscript that addresses the points raised during the second round of the review process.

Please submit your revised manuscript by Mar 01 2026 11:59PM. If you will need more time than this to complete your revisions, please reply to this message or contact the journal office at plosone@plos.org. When you're ready to submit your revision, log on to https://www.editorialmanager.com/pone/ and select the 'Submissions Needing Revision' folder to locate your manuscript file.

- A letter that responds to each point raised by the academic editor and reviewer(s). You should upload this letter as a separate file labeled 'Response to Reviewers'.
- A marked-up copy of your manuscript that highlights changes made to the original version. You should upload this as a separate file labeled 'Revised Manuscript with Track Changes'.
- An unmarked version of your revised paper without tracked changes. You should upload this as a separate file labeled 'Manuscript'.

If applicable, we recommend that you deposit your laboratory protocols in protocols.io to enhance the reproducibility of your results. Protocols.io assigns your protocol its own identifier (DOI) so that it can be cited independently in the future. For instructions see: https://journals.plos.org/plosone/s/submission-guidelines#loc-laboratory-protocols. Additionally, PLOS ONE offers an option for publishing peer-reviewed Lab Protocol articles, which describe protocols hosted on protocols.io. Read more information on sharing protocols at https://plos.org/protocols?utm_medium=editorial-email&utm_source=authorletters&utm_campaign=protocols.

We look forward to receiving your revised manuscript.

Kind regards,

Bin Zhang

Academic Editor

PLOS One

Journal Requirements:

If the reviewer comments include a recommendation to cite specific previously published works, please review and evaluate these publications to determine whether they are relevant and should be cited. There is no requirement to cite these works unless the editor has indicated otherwise.

Please review your reference list to ensure that it is complete and correct. If you have cited papers that have been retracted, please include the rationale for doing so in the manuscript text, or remove these references and replace them with relevant current references. Any changes to the reference list should be mentioned in the rebuttal letter that accompanies your revised manuscript. If you need to cite a retracted article, indicate the article’s retracted status in the References list and also include a citation and full reference for the retraction notice.

[Note: HTML markup is below. Please do not edit.]

Reviewers' comments:

Reviewer's Responses to Questions

Reviewer #1: All comments have been addressed

Reviewer #2: All comments have been addressed

**********

2. Is the manuscript technically sound, and do the data support the conclusions?

Reviewer #1: Yes

Reviewer #2: (No Response)

**********

3. Has the statistical analysis been performed appropriately and rigorously?

Reviewer #1: N/A

Reviewer #2: (No Response)

**********

4. Have the authors made all data underlying the findings in their manuscript fully available?

The PLOS Data policy

Reviewer #1: Yes

Reviewer #2: Yes

**********

5. Is the manuscript presented in an intelligible fashion and written in standard English?

Reviewer #1: Yes

Reviewer #2: Yes

**********

Reviewer #1: Thank you for providing appropriate responses to the editor and reviewers' comments/feedback in this revision.

Reviewer #2: Overall

The study focused on a rare disease (Hemophilia B), matching EHRs (electronic health records) with claims data (prescription/billing data) using tokenization technology and manually verifying the accuracy (due to the small scale). This approach is impossible with a large dataset, and constitutes a novelty.

1. Soundness and Rigor

The study's conclusion (tokenization is useful but lacks data completeness) is valid, but the process by which it was derived is open to debate.

In verifying the accuracy of tokenization, one of the criteria for accuracy (true positive) is whether the claims data contains hemophilia-related medication, test, or diagnostic codes. If tokenization (matching) is successful but no hemophilia-related claims occurred in the claims data during that period (e.g., the patient used stock medication or visited another hospital), there is a risk of misidentifying this as a "false negative." Regarding cases where "linkage is established but disease-related codes are missing," the possibility of a "data completeness issue" should be more clearly distinguished and described in the Methods/Limitations section rather than simply dismissing it as a mismatch.

The validity of the "7-day window" is justified by allowing 7 days for laboratory data matching, but this carries the risk of "overmatching." If a sensitivity analysis could be conducted to show the change in matching rate when the window is changed to 0, 3, or 7 days, the robustness of the 7-day setting would be supported by data.

2. Data Sufficiency and Statistical Support

In the peer review response, the authors claim that "inferential statistics are unnecessary because this is a descriptive study," but this is half right and half insufficient.

N=93 is by no means a small number for rare diseases. It is logical to take advantage of this "small sample size" and emphasize that "manual validation of all cases is a strength." However, even when only descriptive statistics are used, accuracy estimates (e.g., 98% link accuracy) should be accompanied by a 95% confidence interval. Indicating the confidence margin for a 23-case error with a denominator of N=93 is essential for scientific rigor. Rather than simply stating "98%," we recommend calculating and adding a confidence interval, such as "98% (95% CI: XX% YY%)."

3. Bias Assessment

This study exhibits several structural biases. These should be more clearly disclosed in the limitations section.

As for selection bias (digital divide/selection bias), PicnicHealth (the EHR provider) is a platform where patients self-register. Patients who register here may be more IT-literate, health-conscious, and have more severe disease and a greater willingness to manage their condition than the general hemophilia population. Therefore, the high level of EHR data completeness may be due to bias caused by platform characteristics (i.e., a large number of engaged patients). It is dangerous to generalize this to the nature of EHR datasets in general.

Funding Source and Vendor Bias:

The authors are employees of Pfizer, and data was provided by PicnicHealth and Komodo Health. While it is natural to conclude that "EHR and Claims integration is useful (i.e., the usefulness of these services)," caution is needed from a conflict of interest (COI) perspective to ensure that the results are not interpreted in a positive light. While the statement "PicnicHealth provides significantly more complete data..." is included, it would be more neutral to state that "the self-registration nature of EHRs results in higher data density" rather than "PicnicHealth's system is superior."

Visual Aids

The process of data integration through tokenization is complex and difficult to understand through text alone. To facilitate reader understanding, it would be helpful to include or consider adding a conceptual diagram like the one below.

**********

what does this mean?). If published, this will include your full peer review and any attached files.

If you choose “no”, your identity will remain anonymous but your review may still be made public.

**Do you want your identity to be public for this peer review?**  For information about this choice, including consent withdrawal, please see our Privacy Policy

Reviewer #1: No

Reviewer #2: No

**********

[NOTE: If reviewer comments were submitted as an attachment file, they will be attached to this email and accessible via the submission site. Please log into your account, locate the manuscript record, and check for the action link "View Attachments". If this link does not appear, there are no attachment files.]

To ensure your figures meet our technical requirements, please review our figure guidelines: https://journals.plos.org/plosone/s/figures

You may also use PLOS’s free figure tool, NAAS, to help you prepare publication quality figures: https://journals.plos.org/plosone/s/figures#loc-tools-for-figure-preparation.

NAAS will assess whether your figures meet our technical requirements by comparing each figure against our figure specifications.

# Author comment: Author response to Decision Letter 2

**DOI:** 10.1371/journal.pone.0355051.r004

2 Apr 2026

'Response to Reviewers' Word document attached

Attachment Submitted filename: Rebuttal_Letter_PLoSOne_auresp_2.docx

# Peer review: Decision Letter 2

**DOI:** 10.1371/journal.pone.0355051.r005

**Authors:** Bin Zhang

20 Apr 2026

Dear Dr. Stan,

Thank you for submitting your manuscript to PLOS ONE.

Reviewer 2 still has significant concerns about the manuscript, many of which stem from different opinions/interpretations of the data. Since the manuscript has gone through two rounds of reviews, and the “Limitations” section has become unusually long and unwieldy. To not endlessly prolong the review process, I suggest that you focus on responding to the following points:

1. A major dispute is whether 95% confidence interval calculation should be included. I agree that this is unnecessary and may be misleading for the actual observations, instead of estimates. Please add “in our data” following “achieved a high linkage accuracy rate (93–98%)” in line 373.

2. Regarding the “7-day matching window” comment, can you compare the matching rates at 0, 3, 7 and 15 days as suggested by the reviewer? If you have good reasons to skip this analysis, such as feasibility issues, please elaborate.

3. Regarding the reviewer’s concern that PicnicHealth is a patient self-registration platform, does the patient have a role beyond registration? The manuscript mentioned in passing that healthcare providers may also enter data into this EHR. It may be helpful to specify the roles of patients and healthcare providers in PicnicHealth data accumulation.

4. Please respond to the comment that “Overemphasizing the "98%" figure in the conclusion is an overstatement that exceeds the limitations of the study design”.

Please submit your revised manuscript by Jun 04 2026 11:59PM. If you will need more time than this to complete your revisions, please reply to this message or contact the journal office at plosone@plos.org. When you're ready to submit your revision, log on to https://www.editorialmanager.com/pone/ and select the 'Submissions Needing Revision' folder to locate your manuscript file.

- A letter that responds to each point raised by the academic editor and reviewer(s). You should upload this letter as a separate file labeled 'Response to Reviewers'.
- A marked-up copy of your manuscript that highlights changes made to the original version. You should upload this as a separate file labeled 'Revised Manuscript with Track Changes'.
- An unmarked version of your revised paper without tracked changes. You should upload this as a separate file labeled 'Manuscript'.

If you would like to make changes to your financial disclosure, please include your updated statement in your cover letter. Guidelines for resubmitting your figure files are available below the reviewer comments at the end of this letter.

If applicable, we recommend that you deposit your laboratory protocols in protocols.io to enhance the reproducibility of your results. Protocols.io assigns your protocol its own identifier (DOI) so that it can be cited independently in the future. For instructions see: https://journals.plos.org/plosone/s/submission-guidelines#loc-laboratory-protocols. Additionally, PLOS ONE offers an option for publishing peer-reviewed Lab Protocol articles, which describe protocols hosted on protocols.io. Read more information on sharing protocols at https://plos.org/protocols?utm_medium=editorial-email&utm_source=authorletters&utm_campaign=protocols.

As the corresponding author, your ORCID iD is verified in the submission system and will appear in the published article. PLOS supports the use of ORCID, and we encourage all coauthors to register for an ORCID iD and use it as well. Please encourage your coauthors to verify their ORCID iD within the submission system before final acceptance, as unverified ORCID iDs will not appear in the published article. *Only*  the individual author can complete the verification step; PLOS staff *cannot*  verify ORCID iDs on behalf of authors.

We look forward to receiving your revised manuscript.

Kind regards,

Bin Zhang

Academic Editor

PLOS One

**Journal Requirements:**

1. If the reviewer comments include a recommendation to cite specific previously published works, please review and evaluate these publications to determine whether they are relevant and should be cited. There is no requirement to cite these works unless the editor has indicated otherwise.

2. Please review your reference list to ensure that it is complete and correct. If you have cited papers that have been retracted, please include the rationale for doing so in the manuscript text, or remove these references and replace them with relevant current references. Any changes to the reference list should be mentioned in the rebuttal letter that accompanies your revised manuscript. If you need to cite a retracted article, indicate the article’s retracted status in the References list and also include a citation and full reference for the retraction notice.

Reviewers' comments:

Reviewer's Responses to Questions

**Comments to the Author**

Reviewer #2: All comments have been addressed

**********

2. Is the manuscript technically sound, and do the data support the conclusions?

Reviewer #2: Partly

**********

3. Has the statistical analysis been performed appropriately and rigorously?

Reviewer #2: N/A

**********

4. Have the authors made all data underlying the findings in their manuscript fully available?

The PLOS Data policy

Reviewer #2: No

**********

5. Is the manuscript presented in an intelligible fashion and written in standard English?

Reviewer #2: Yes

**********

Reviewer #2: Overall

By focusing on a unique population with a rare disease (hemophilia B), the accuracy of data integration using tokenization technology (EHR and claims data) was verified through a "manual review of all cases (N=93)." This is a very clever approach and possesses a uniqueness that would be impossible with large datasets. It provides practical insights into real-world data (RWD) integration methods and has high relevance and value in the fields of medical informatics and rare disease research.

Even after the authors' rebuttal, a significant lack of "rigor" remains.

The authors argue that "this study is descriptive, therefore inferential statistics are unnecessary and could mislead readers," and explicitly reject the "calculation of 95% confidence intervals (CI)" pointed out in the previous review. However, presenting only accuracy (point estimates) such as 98% or 93% with a limited sample size of N=93 is a serious lack of scientific rigor.

Descriptive percentages that do not indicate the range of estimation (margin of error) for the population can actually lead readers to the misconception (overconfidence) that "near-100% accuracy is always guaranteed." While the conclusion itself (tokenization is feasible and reliable in rare diseases) is generally valid, the deliberate abandonment of statistical robustness to support that accuracy is a major problem.

The data size of N=93 is sufficient for a pilot study of rare diseases, but insufficient for generalizing the results. Furthermore, the handling of the following biases remains incomplete:

The authors added a sentence about "patient-mediated EHR aggregation platform" in the Limitations section and mentioned "engagement-related selection bias." However, there is a lack of in-depth consideration of the impact of the fundamental structural bias—that patients who self-register with PicnicHealth have high IT literacy and are very proactive in managing their own health—on the conclusion of data completeness (the overwhelming density of EHR data).

There is a conflict of interest as the author is a Pfizer employee and is evaluating licensed platforms (PicnicHealth, Komodo Health). While the wording was revised to be more neutral, stating "it's not about which data source is superior, but the difference in architecture," there is still a tendency to overemphasize the usefulness of the platforms in the interpretation of the results.

This study does not use complex mathematical models, so there is no omission of advanced mathematical formulas. However, as mentioned above, it completely lacks "basic statistical calculations for assessing accuracy uncertainty (calculation of confidence intervals)."

Furthermore, regarding the "7-day matching window," the author justifies it in the text as "a reasonable period considering administrative delays," but the calculation process of a sensitivity analysis showing how this affected overmatching and missing matches (e.g., numerical changes in matching rates at 0, 3, 7, and 14 days) is not shown, remaining merely an "adoption of assumptions."

Because encrypted tokens are used, there is no true "gold standard" (direct personal identification by real name, etc.). The use of "clinical record matching (diagnosis of hemophilia and medication records)" as a proxy metric to create the correct label is a realistic and optimal design from a privacy protection perspective.

However, the design for clearly distinguishing between cases where "the link is successful but billing data simply did not occur during that period (data incompleteness)" and cases where "the link itself is incorrect (linkage error)" is weak (e.g., accuracy validation limited to subgroups with complete closed billing data), leaving ambiguity in the interpretation of the results.

Regarding the conclusion that "tokenization achieved up to 98% accuracy," the validity of this figure is questionable. While the "98%" figure is based on a combination of ZIP code matching and clinical data matching, due to the nature of encrypted data, this is merely "confirmation of consistency," not "confirmation of identity." Overemphasizing the "98%" figure in the conclusion is an overstatement that exceeds the limitations of the study design.

Regarding the conclusion that "EHR data is more complete than claims data (EHR 89100% vs Claims 44100%)":

While this conclusion is highly valid, it can also be seen as a self-fulfilling prophecy when comparing a platform where patients intentionally aggregate their medical records (PicnicHealth) with passively generated, fragmented claims data (Komodo). It serves as a valid path to the final conclusion that "tokenization is useful for complementing these two types of data."

AI tools were used to edit the language of this decision notification. However, in accordance with this journal’s policy regarding the use of AI tools in peer review, no AI tools were used in the evaluation of the submitted manuscript. The editor in charge did not upload the author’s personal information or the manuscript content to any AI tools.

**********

what does this mean?). If published, this will include your full peer review and any attached files.

If you choose “no”, your identity will remain anonymous but your review may still be made public.

**Do you want your identity to be public for this peer review?**  For information about this choice, including consent withdrawal, please see our Privacy Policy

Reviewer #2: No

**********

[NOTE: If reviewer comments were submitted as an attachment file, they will be attached to this email and accessible via the submission site. Please log into your account, locate the manuscript record, and check for the action link "View Attachments". If this link does not appear, there are no attachment files.]

To ensure your figures meet our technical requirements, please review our figure guidelines: https://journals.plos.org/plosone/s/figures

You may also use PLOS’s free figure tool, NAAS, to help you prepare publication quality figures: https://journals.plos.org/plosone/s/figures#loc-tools-for-figure-preparation.

NAAS will assess whether your figures meet our technical requirements by comparing each figure against our figure specifications.

# Author comment: Author response to Decision Letter 3

**DOI:** 10.1371/journal.pone.0355051.r006

1 Jul 2026

Dear Editors and Reviewers,

We thank the Editor and Reviewer 2 for the additional thoughtful comments and suggestions. We have carefully revised the manuscript and provide our point-by-point responses below.

Comment 1:

‘A major dispute is whether 95% confidence interval calculation should be included. I agree that this is unnecessary and may be misleading for the actual observations, instead of estimates. Please add “in our data” following “achieved a high linkage accuracy rate (93–98%)” in line 373.’

Response:

We thank the Editor for this clarification and agree that confidence intervals are unnecessary in the context of this descriptive methodological evaluation. As suggested, we revised the conclusion to clarify that these values represent observed findings within our study sample rather than inferential population estimates. The sentence was revised to:

“In our data, the tokenization process demonstrated high linkage accuracy (93% or 98%, depending on the validation variables assessed).” This revision also clarifies that the reported values reflect different validation approaches rather than a statistical interval.

Comment 2:

‘Regarding the “7-day matching window” comment, can you compare the matching rates at 0, 3, 7 and 15 days as suggested by the reviewer? If you have good reasons to skip this analysis, such as feasibility issues, please elaborate.’

Response:

We appreciate this suggestion and agree that evaluation of alternative matching windows would be informative. However, conducting additional sensitivity analyses was not feasible within the current revision process because the analyses were originally conducted within a third-party hosted environment that is no longer accessible following completion of the project collaboration.

To address this concern, we further clarified in the Limitations section that alternative temporal definitions may yield different completeness estimates and that future studies in larger cohorts should formally evaluate the impact of varying window definitions on matching performance.

Comment 3:

‘Regarding the reviewer’s concern that PicnicHealth is a patient self-registration platform, does the patient have a role beyond registration? The manuscript mentioned in passing that healthcare providers may also enter data into this EHR. It may be helpful to specify the roles of patients and healthcare providers in PicnicHealth data accumulation.’

Response:

We appreciate the opportunity to clarify this point and revised the manuscript accordingly. Patients may enroll into the PicnicHealth platform; however, the EHR data itself is passively collected from existing medical records by the PicnicHealth abstractors. Neither patients nor healthcare providers directly enter clinical information into the PicnicHealth platform for purposes of this study. More information about the PicnicHealth platform and the abstraction process can be found here: Picnichealth.com

We revised the manuscript to clarify that PicnicHealth validates diagnoses using medical records and that the platform contains data derived from the underlying medical records obtained from healthcare providers.

Comment 4:

‘Please respond to the comment that “Overemphasizing the ‘98%’ figure in the conclusion is an overstatement that exceeds the limitations of the study design”.’

Response:

We appreciate this comment and revised the conclusion to avoid overstating the findings. Specifically, we clarified that the linkage accuracy values reflect observations within our study sample and revised the wording to present the findings in a more descriptive and methodologic manner. This wording better contextualizes the findings within the limitations of the study design.

Additional Revision:

In response to the Editor’s suggestion, we also consolidated the Limitations section to improve readability while retaining the major methodological considerations and contextual points raised during peer review.

We thank the Editor and Reviewer for their valuable feedback, which has helped improve the clarity and methodological transparency of the manuscript.

Attachment Submitted filename: Response to Reviewers_v2.docx

# Editor report: Decision Letter 3

**DOI:** 10.1371/journal.pone.0355051.r007

**Authors:** Bin Zhang

16 Jul 2026

Evaluation of the Completeness of a Medical Record Dataset Linked to Administrative Claims through Tokenization in Patients with Hemophilia B

PONE-D-25-21079R3

Dear Dr. Stan,

We’re pleased to inform you that your manuscript has been judged scientifically suitable for publication and will be formally accepted for publication once it meets all outstanding technical requirements.

Within one week, you’ll receive an e-mail detailing the required amendments. When these have been addressed, you’ll receive a formal acceptance letter and your manuscript will be scheduled for publication.

An invoice will be generated when your article is formally accepted. Please note, if your institution has a publishing partnership with PLOS and your article meets the relevant criteria, all or part of your publication costs will be covered. Please make sure your user information is up-to-date by logging into Editorial Manager at Editorial Manager® and clicking the ‘Update My Information' link at the top of the page. For questions related to billing, please contact billing support.

If your institution or institutions have a press office, please notify them about your upcoming paper to help maximize its impact. If they’ll be preparing press materials, please inform our press team as soon as possible -- no later than 48 hours after receiving the formal acceptance. Your manuscript will remain under strict press embargo until 2 pm Eastern Time on the date of publication. For more information, please contact onepress@plos.org.

Kind regards,

Bin Zhang

Academic Editor

PLOS One

# Editor report: Acceptance letter

**DOI:** 10.1371/journal.pone.0355051.r008

**Authors:** Bin Zhang

PONE-D-25-21079R3

PLOS One

Dear Dr. Stan,

I'm pleased to inform you that your manuscript has been deemed suitable for publication in PLOS One. Congratulations! Your manuscript is now being handed over to our production team.

At this stage, our production department will prepare your paper for publication. This includes ensuring the following:

* All references, tables, and figures are properly cited

* All relevant supporting information is included in the manuscript submission,

* There are no issues that prevent the paper from being properly typeset

You will receive further instructions from the production team, including instructions on how to review your proof when it is ready. Please keep in mind that we are working through a large volume of accepted articles, so please give us a few days to review your paper and let you know the next and final steps.

Lastly, if your institution or institutions have a press office, please let them know about your upcoming paper now to help maximize its impact. If they'll be preparing press materials, please inform our press team within the next 48 hours. Your manuscript will remain under strict press embargo until 2 pm Eastern Time on the date of publication. For more information, please contact onepress@plos.org.

You will receive an invoice from PLOS for your publication fee after your manuscript has reached the completed accept phase. If you receive an email requesting payment before acceptance or for any other service, this may be a phishing scheme. Learn how to identify phishing emails and protect your accounts at https://explore.plos.org/phishing.

If we can help with anything else, please email us at customercare@plos.org.

Thank you for submitting your work to PLOS One and supporting open access.

Kind regards,

PLOS One Editorial Office Staff

on behalf of

Dr. Bin Zhang

Academic Editor

PLOS One
